# Youth 2020 — Preventing Another Lost Generation?

**DOI:** 10.1007/s10272-020-0932-y

**Published:** 2020-12-01

**Authors:** Sue Maguire

**Affiliations:** Institute for Policy Research, University of Bath, Claverton Down, BA2 7AY Bath, UK

## Abstract

The potential scarring effects of long-term youth unemployment and social disengagement have for many years challenged policymakers to develop successful and sustainable interventions.

Across the EU and internationally, as the second wave of COVID-19 takes hold, the economic impact emanating from the pandemic is evident. This includes widespread increases in unemployment and economic inactivity. As was the case in previous times of economic shock, such as in the 1980s, 1990s, and, in particular, in the economic recession following the 2008 financial crash, it is already evident that young people will be disproportionately affected. This has reignited fears that there will emerge a 'lost generation' of young people, who will be denied access to good quality and sustainable job opportunities as they transition into the labour market.

The potential scarring effects of long-term youth unemployment and social disengagement have for many years challenged policymakers to develop successful and sustainable interventions (Bell and Blanchflower, 2011). For example, in the UK, evidence from the Commission on Youth Unemployment showed that unemployed young people aged 16-24 years were more likely to spend longer periods out of work throughout their lives, be paid less when in work (Macmillan, 2012), have poorer mental and physical wellbeing and be involved in criminal activity (Bell and Blanchflower, 2011). At the same time, the costs to the public purse of managing the economic and social consequences of inactivity among young people cannot be ignored.

This article seeks to develop a better understanding of the effectiveness of a range of youth employment programmes, which have been tried and tested to address the needs of different groups of young people who face barriers to accessing the labour market. In addition, they are designed to reduce youth unemployment, as well as (in some cases) economic inactivity.

## Why are young people so vulnerable?

Young people's vulnerability to higher than average rates of unemployment or redundancy is often attributed to their disadvantaged or precarious position in the labour market. Firstly, in terms of a labour 'queue', young people's limited labour market experience and/or their perceived inadequate skill levels render them less likely to be recruited at times of high unemployment. Secondly, structural changes in the labour market, emanating from technological advances and sectoral shifts are adversely affecting young people's job opportunities. For example, a survey conducted in May 2020 about the impact of COVID-19 in the UK found that "one-third of 18-24-year old employees (excluding students) have lost their jobs or been furloughed, compared to one-in-six prime-age adults" (Gustafsson, 2020, 1).

Moreover, the industrial sectors that will be most affected by job cuts in 2020 and possibly beyond, notably, non-food retail, hospitality, travel, the arts and entertainment are those that employ a large number of non-graduates. Young people are further disadvantaged by being among any initial redundancies due to their comparatively shorter period of employment in a company than their co-workers (Henehan, 2020).

## Policies to address youth unemployment

There is clearly no one 'silver bullet' response to escalating levels of youth unemployment. According to O'Higgins (2001), when developing potential policies to address youth unemployment, key considerations to be taken into account are:

- the economic context
- an appraisal and understanding of the target group
- identifying parts of the economy that have potential for growth.

In most cases, some form of active labour market policy (ALMP) has been implemented to prevent and reduce unemployment, including for young people who are unemployed or not in education, employment or training (NEET). Different ALMPs are designed to reduce various types of unemployment, cyclical unemployment, structural unemployment, regional disparities – together with measures that support disadvantaged groups which may have additional barriers. As discussed in Duell (2012), they also vary substantially between 'work first' and 'train first' strategies, depending on whether the focus is on a rap-

**Sue Maguire**, University of Bath, UK.

id reduction of unemployment (work first) and/or a more sustainable integration into the labour market (train first).

Eichhorst et al. (2016) differentiated ALMPs into five types:

- job-search assistance
- training programmes
- subsidised employment
- direct job creation and public employment programmes
- start-up subsidies, self-employment assistance and support.

### Job-search assistance

In the past, job-search assistance measures that focused on early intervention to encourage targeted and informed job searches were considered to be as effective as more expensive programmes such as job creation and job subsidies (OECD, 1993). More recent research has confirmed the efficacy of job-search assistance and monitoring programmes in terms of leading to accessing employment, as well as their cost-effectiveness and capability of working in different occupational settings (Caliendo and Schmidl, 2016). By being introduced as soon as an individual becomes unemployed, they reduce the risk of longer-term unemployment.

Concerns that have been raised about job-search assistance programmes are a) the possibility of some young people accepting any form of work, including precarious employment, because they fear that they will be sanctioned or b) the possibility that they will be discouraged from registering as unemployed and withdraw from the labour market. In addition, research identified a reduction in the subsequent earnings of participants in some countries, such as Canada (O'Higgins, 2001).

In substantive and successful programmes such as the European Youth Guarantee and the New Deal for Young People in the UK, a job-search assistance element has been included to accompany more general training and employment elements.

### Training programmes

Training programmes to support young people's transitions into the labour market have been introduced in many countries over a considerable period of time. Overall, however, their impact has been found to be insignificant, or even negative, notably when classroom-based training was provided (Caliendo and Schmidt, 2016, 17). As a response to mass unemployment, training programmes have been criticised for offering only a brief interlude of being 'warehoused', without enhancing qualification attainment or progression to the labour market (Keep, 1986).

Furthermore, evaluations of training programmes targeted at disadvantaged young people have tended to focus predominantly on 'hard' outcomes, such as numbers becoming employed, rather than taking into consideration 'soft' outcomes, such as 'distance travelled', and therefore indicate poor programme performance (Martin and Grubb, 2001). This leads to the recruitment of young people who are immediately employable, detracting attention from young people who require sustained and supported intervention before they can access employment.

While it has been evidenced that apprenticeship programmes can assist access to jobs in times of recession, even in countries with well-established apprenticeship systems, such as Germany, Austria, Switzerland and Luxembourg, employers can be reluctant to recruit young people, especially if they have no or low-level qualifications (Scarpetta et al., 2010). Such reluctance is likely to be exacerbated by the constraints of COVID-19, with social distancing restrictions making it more difficult to accommodate and train new recruits.

A more positive note was struck by Duell (2012), who suggested that a positive impact of pre-vocational programmes for low-skilled young people had been prevalent where well-developed dual training systems were in place. These programmes incorporated an individualised approach involving vocational guidance, basic skills training and work-related training. Furthermore, Duell (2012) concluded that such an individualised approach was a key prerequisite for these programmes to be effective.

### Subsidised employment

Subsidised employment may include wage subsidies or wage cost subsidies, both of which are found to have a positive impact on employment outcomes, especially if they are well targeted towards disadvantaged groups, including young people (Duell, 2012). They can also play an important role in helping maintain the attachment of young people to the labour market and offer employers training subsidies, as well as incentives to sustain their recruitment.

However, while evaluations of whole population studies have concluded that wage subsidies can have a positive impact in the short run, substantial deadweight and substitution effects – in terms of employers' behaviour – have been present (Martin and Grubb, 2001).

### Direct job creation and public employment programmes

Job creation and public employment programmes provide young people with a degree of attachment to the labour market, especially during a recession. When combined with vocational training, they become more expensive to implement. A macroeconomic study using analysis of a database with repeated observations for all EU member states for the 1998-2012 period concluded that older groups in the youth cohort aged 20-24 years were more likely than younger groups to benefit from job creation programmes (Speckesser et al., 2019). This was attributed to older groups having experienced prolonged detachment from the labour market, as well as from education and training intervention. Other studies have shown that public works can help more disadvantaged groups (including young people), as they provide participants with some form of income in the absence of little or no welfare support (Kuddo, 2012).

### Start-up subsidies, self-employment assistance and support

Policymakers in many countries favour the implementation of programmes that encourage young people to become self-employed, usually as a subsidiary component of an overall attempt to reduce youth unemployment. However, take-up rates tend to be low, due to young people's shortage of skills and experience, and a lack of funding and support (OECD/European Commission, 2020).

Typically, self-employment programmes may comprise one or several of the following elements (O'Higgins, 2001, 125):

- promoting and introducing the self-employment option
- training in skills development
- mentorship
- financial support
- access to workspace
- business expansion support
- networking.

More recent assessment, while pointing to the need for a combination of these components to be offered, questions whether the emphasis should be weighted in favour of financial support or the development of entrepreneurial skills. A caveat made here is that, in view of the substantial increase in the number of youth entrepreneurship programmes, more evidence is required (OECD/European Commission, 2020).

### Multi-element programmes

As well as introducing individual ALMPs, policymakers have devised multi-element programmes to form an integrated offer. This may include a combination of job search and counselling, education and training, wage incentives and job creation. The most recent and powerful evidence, which has assessed the extent to which labour market interventions have successfully improved young people's employment outcomes, concluded that programmes which integrate a number of interventions and services are more likely to be successful, in particular in low- and middle-income countries (Kluve et al., 2019). Crucially, being underpinned by effective and efficient profiling and follow-up systems was found to be pivotal to their success.

## The effectiveness of youth employment programmes

Echoing O'Higgins' (2001) identification of what needs to be taken into account when devising appropriate policies, it is necessary to consider:

*The state of the economy*. This means acknowledging that what works will vary, dependent on the prevailing economic conditions and existing infrastructure arrangements. For example, employment subsidies can be effective at times of recession, as they can move young people who are 'labour market ready' into employment very quickly and at volume. However, these programmes are prone to deadweight and substitution effects. Youth guarantees also have the capacity to ensure young people are given an offer of employment or training and can reduce the risk of young people being locked into long-term unemployment/economic inactivity.

*The target group of young people the policies are seeking to support*. To be successful, policies need to be tailored to meet the needs of the target group, rather than attempting to impose a rigid system encompassing all young people within a certain age group. This is particularly pertinent when the impact of COVID-19 means that the volume and characteristics of young people becoming unemployed may differ from those affected by economic recessions. It has been suggested that, during an economic recession, an emphasis on targeting could enhance the positive impact of an ALMP (Card et al., 2015; Kluve et al., 2019). Furthermore, by taking situational and economic conditions into consideration, ALMPs focusing on young people have a more positive impact (Caliendo and Schmidl, 2016, 17).

Shortcomings of ALMPs in relation to their impact on young people include:

- The propensity, particularly for training and subsidised employment programmes, to demonstrate large deadweight, substitution and displacement effects. In making such calculations, it is important to differentiate between programmes that are designed to have an immediate impact on reducing levels of unemployment and those that seek to provide a more sustainable transition to the labour market through training (Duell, 2012);

- Their tendency to focus on 'work ready' young people, at the expense of 'harder to help' and 'harder to reach' groups;

- A lack of evidence about 'what works and at what cost', notably a 'short-sighted' emphasis on measuring job outcomes, rather than on the 'distance travelled' by individuals and the impact which training and/or work experience may have on their attachment to the labour market (Caliendo and Schmidl, 2016);

- The costs of programmes often being vague, inconsistent and incomplete.

Overall, ALMPs are unlikely to work for the most disadvantaged groups unless accompanied by re-engagement strategies.

Characteristics of programmes which have been found to be effective include:

- being closely targeted rather than generic
- outreach services that extend and encourage engagement and participation
- the provision of intensive support for young people furthest away from the labour market
- having pre-vocational programmes for low-skilled young people
- multi-element programmes forming an integrated offer, which may include a one-stop-shop to provide benefit payments, career guidance and counselling, assistance with job search and placements, referral to further training and other welfare services
- having effective and efficient profiling and follow-up systems.

## Conclusions

The economic impact of the COVID-19 pandemic will result in mass or increased youth unemployment in many countries. In recent decades, there have been many programmes and interventions to help prevent and stem the flow of high incidences of youth unemployment and inactivity. However, it is crucial that their performance is assessed before lurching into immediate policy responses. Research evidence tells us that introducing 'knee-jerk' policies without understanding the needs of young people and the labour market can be both costly and damaging.

Programme evaluation has highlighted the importance of targeting. Achieving this objective is dependent upon having tracking systems in place that can produce robust, reliable and efficient data on young people's intended and actual destinations, alongside accurate labour market information, which is sensitive to the needs of regional and local labour markets.

It is also vitally important that programmes and interventions are designed to recognise and meet the needs of all groups of young people, including the hardest to help/reach and young people who are defined as economically inactive, due to their caring responsibilities or ill health.

Establishing or maintaining services that facilitate early identification and early intervention are critical components that improve the effectiveness of ALMPs. This process is enhanced by offering young people who require support an individualised and person-centred approach. Moreover, on-programme support and follow-up once young people enter the labour market are also likely to improve sustainability impacts.

The evidence suggests that a one-size-fits-all approach simply will not work and that a range of interventions, while costly, will be needed to meet the needs of a diverse population and prevent the risk of a 'lost generation'. Moreover, while there will be assertions that the cost of delivering such ambitions may be prohibitive, a counter argument must be that the cost of failing successive generations of young people remains unacceptable.
